# d,l-Methadone causes leukemic cell apoptosis via an OPRM1-triggered increase in IP3R-mediated ER Ca^2+^ release and decrease in Ca^2+^ efflux, elevating [Ca^2+^]_i_

**DOI:** 10.1038/s41598-020-80520-w

**Published:** 2021-01-13

**Authors:** JungKwon Lee, Jesusa L. Rosales, Hee-Guk Byun, Ki-Young Lee

**Affiliations:** 1 Department of Cell Biology and Anatomy, Arnie Charbonneau Cancer, Alberta Children’s Hospital Research Institutes, Cumming School of Medicine, University of Calgary, Calgary, AB T2N 4N1 Canada; 2 Department of Marine Biotechnology, Gangneung-Wonju National University, Gangneung, South Korea

**Keywords:** Diseases, Medical research, Oncology

## Abstract

The search continues for improved therapy for acute lymphoblastic leukemia (*a*LL), the most common malignancy in children. Recently, d,l-methadone was put forth as sensitizer for *a*LL chemotherapy. However, the specific target of d,l-methadone in leukemic cells and the mechanism by which it induces leukemic cell apoptosis remain to be defined. Here, we demonstrate that d,l-methadone induces leukemic cell apoptosis through activation of the mu1 subtype of opioid receptors (OPRM1). d,l-Methadone evokes IP3R-mediated ER Ca^2+^ release that is inhibited by OPRM1 loss. In addition, the rate of Ca^2+^ extrusion following d,l-methadone treatment is reduced, but is accelerated by loss of OPRM1. These d,l-methadone effects cause a lethal rise in [Ca^2+^]_i_ that is again inhibited by OPRM1 loss, which then prevents d,l-methadone-induced apoptosis that is associated with activation of calpain-1, truncation of Bid, cytochrome C release, and proteolysis of caspase-3/12. Chelating intracellular Ca^2+^ with BAPTA-AM reverses d,l-methadone-induced apoptosis, establishing a link between the rise in [Ca^2+^]_i_ and d,l-methadone-induced apoptosis. Altogether, our findings point to OPRM1 as a specific target of d,l-methadone in leukemic cells, and that OPRM1 activation by d,l-methadone disrupts IP3R-mediated ER Ca^2+^ release and rate of Ca^2+^ efflux, causing a rise in [Ca^2+^]_i_ that upregulates the calpain-1-Bid-cytochrome C-caspase-3/12 apoptotic pathway.

## Introduction

Acute lymphoblastic leukemia (*a*LL) is a malignancy of B- or T-lymphoblasts that mostly affects children^1^. It comprises over a quarter of all pediatric cancers and causes most of the cancer deaths among children^2^. While newer targeted therapies for *a*LL have been developed, their effectiveness is enhanced when used in combination with chemotherapy, which remains to be the major treatment for *a*LL. Chemotherapy drugs kill leukemic cells by inducing apoptosis through blockade of nucleic acid synthesis or direct targeting of DNA, interfering with protein synthesis or inhibiting mitosis, respectively^3^. Recently, the synthetic opioid, d,l-methadone, was hailed as an antineoplastic agent, particularly for leukemia^4–6^ and glioblastoma^4,7^. It was also shown to sensitizes leukemia^5,6^ and glioblastoma cells^7^ to doxorubicin treatment.

d,l-Methadone was first developed for the stabilization and maintenance of patients suffering from addiction to other opioids such as heroin, oxycodone or fentanyl^8^. It is also used as an analgesic for the management of chronic pain such as in cancer^9^. The pharmacological effects of d,l-methadone have been associated with its interaction with Fas cell death receptors^10^, toll-like receptors^11^, 3β4 nicotinic acetylcholine receptors^12^ and *N*-methyl-d-aspartate receptors^13^, but primarily with opioid G-protein-coupled receptors (GPCRs)^14^. Thus far, four types of opioid receptors have been cloned: the classical mu (μ), kappa (κ) and delta (δ) opioid receptors, and the nociceptin/orphanin FQ receptor (aka opioid receptor-like receptor)^15^. Three subtypes of the mu opioid receptor have been proposed: mu1, mu2, and morphine-6β-glucuronide (M6G)^16^. Although these opioid receptor subtypes have high homology, each receptor poses distinct localization, agonist selectivity, and transcriptional regulation^15^.

Activation of opioid GPCRs induces conformational change in the receptor that facilitates the exchange of GDP for GTP on G_α_, leading to uncoupling from G_βγ_^17^. Uncoupled GTP-bound G_α_ and G_βγ_ dimer regulate diverse physiological functions including apoptosis^5^ through stimulation of their downstream effectors such as cyclic adenosine monophosphate (cAMP) and Ca^2+^^18^. As with other GPCRs, opioid receptors bind to G_α_ subunits, including members of the G_αi/o_ family^19,20^. G_*α*i/o_ subunit activation by opioid receptors has been shown to decrease intracellular cAMP levels^21,22^ induced by the adenylyl cyclase activator, forskolin^23^. On the other hand, the G_βγ_ subunit mediates opioid-induced stimulation of phospholipase C β (PLCβ); none of the pertussis toxin (PTX)-sensitive G_α_ subunits can activate PLCβ by themselves^24,25^. In neuroblastoma cells, G_βγ_ subunit activation of PLCβ is linked to opioid-induced ER Ca^2+^ release^26^. This is consistent with the finding that opioid receptor stimulation by opioids causes a surge in [Ca^2+^]_i_ in leukocytes^22,27^, pituitary^28^ and neuroblastoma cells^26^ through G_βγ_, which is now recognized to stimulate PLC, which in turn cleaves phosphatidylinositol 4,5-bisphosphate (PIP2) into diacylglycerol (DAG) and inositol 1,4,5-trisphosphate (IP3). IP3 binds to the IP3 receptor (IP3R), triggering the opening of the IP3R Ca^2+^ channels and release of Ca^2+^ from internal stores to the cytosol. Along these lines, it is not surprising that mu opioid receptors have been implicated in intracellular Ca^2+^ homeostasis in neuroblastoma^29^ and pituitary cells^28^. Thus, as with other receptors that exploit G_αi_ and G_βγ_ for signal transduction, opioid GPCRs act via the adenylyl cyclase/cAMP- and Ca^2+^-mediated pathways.

The finding that d,l-methadone causes opioid receptor-mediated cAMP downregulation in leukemic cells^5^ is consistent with the fact that G_αi_ blocks adenylyl cyclase activity, causing reduced intracellular [cAMP]^17^ that leads to apoptosis through activation of caspases^30^. While d,l-methadone is characterized as an opioid receptor agonist^5^, the specific opioid receptor target and molecular mechanisms of d,l-methadone-induced leukemic cell death remains to be defined.

By unbiased genome-wide retroviral RNAi screening and knockdown studies, we recently identified the mu1 subtype of opioid receptors (opioid receptor mu1: OPRM1) as a novel resistance biomarker for l-asparaginase, a fundamental component of chemotherapy regimens for *a*LL^31^. We found that OPRM1 is required for l-asparaginase to induce apoptosis and that loss of OPRM1 leads to l-asparaginase resistance^31^. This finding together with those described above prompted us to investigate whether d,l-methadone specifically targets OPRM1 to induce leukemic cell apoptosis, and if so, determine whether OPRM1-mediated d,l-methadone-induced apoptosis occurs through aberrations in [Ca^2+^]_i_.

Using POETIC2 *a*LL cells (*) stably infected with retrovirus carrying an empty pRS vector (*+pRS) or pRS-sh*OPRM1* (*+pRS-sh*OPRM1*), we demonstrate that d,l-methadone induces leukemic cell apoptosis by targeting OPRM1 to increase Ca^2+^ release from the endoplasmic reticulum (ER) through the IP3R Ca^2+^ channels and to reduce the rate of intracellular Ca^2+^ efflux, evoking a lethal rise in [Ca^2+^]_i_.

## Materials and methods

### Materials

The Opti-MEM reduced serum media, fetal bovine serum, penicillin–streptomycin, Fura-2 AM, Fluo-4 AM, Mag-Fluo-4 AM, and the Annexin V-FITC staining Kit were from ThermoFisher Scientific (Burlington, ON, Canada). l-asparaginase (ASNase, ab73439) and 2,2-Bis(2-aminophenoxy)ethane-N,N,N′,N′-tetraacetic acid tetrakis (acetoxymethyl ester) (BAPTA-AM) were from Abcam (Toronto, ON, Canada). 2,5-Di-tert-butylhydroquinone (TBHQ) and tetracaine (Tet) were from Sigma (Oakville, ON, Canada). Xestospongin-C (XeC) was from Bio-Techne (Oakville, ON, Canada). Antibodies to OPRM1 (D-12), Bid (B-3), caspase-3 (E-8), caspase-12 (1611), poly(ADP-ribose) polymerase-1 (PARP1, F-2), GAPDH (0411), calpain-1 (6C-12), cytochrome C (H-104), voltage-dependent anion-selective channel 1 (VDAC-1, B-6), tubulin (D-10) and actin (I-19) were from Santa Cruz Biotech. (Dallas, TX, USA). The antibody against cleaved caspase-3 (D1–75) was from Cell Signaling (Whitby, ON). Trypan blue was from Life Technologies (Burlington, ON, Canada). Alamar blue was from Life Technologies (ON, Canada). Calpeptin was from Calbiochem, CA, USA.

### Methods

We confirm that all methods described in this manuscript were carried out in accordance with relevant guidelines and regulations. All experiments followed the University of Calgary’s biosafety guidelines.

### Cell culture

POETIC2 parental cells (*) were derived from 14-year-old pre-B acute lymphoblastic leukemia patient. Generation of POETIC2 cells (*) stably infected with retrovirus carrying a pRS empty vector (*+pRS) or pRS-sh*OPRM1* (*+pRS-sh*OPRM1*) was described previously^31^. POETIC 2 cells were cultured in Opti-MEM, containing 10% FBS and 100 μg/ml penicillin–streptomycin, at 37 °C in 5% CO_2_.

### Determination of surviving cell population

Surviving population of cells (1 × 10^4^ cells/well in 96 well plates) treated with different concentrations of d,l-methadone for 24 h were quantified using Alamar blue assay following the manufacturer’s protocol.

### Measurement of Ca^2+^

(i) To measure resting [Ca^2+^]_i_, the method described by Grynkiewicz et al.^32^ was followed. Briefly, cells (5 × 10^5^) loaded with 5 μM Fluo-4 AM in Ca^2+^-free KRH buffer at 37 °C for 30 min were washed with Ca^2+^-free KRH buffer. Resting [Ca^2+^]_i_ was measured every 2 s at 485_ nm_ excitation/530 nm emission using a Shimadzu RF 5301PC spectrofluorometer. F_max_ value was obtained after treatment with 0.02% saponin for 30 s and addition of 2 µM CaCl_2_ four times. F_min_ value was measured upon addition of 4 mM EDTA. [Ca^2+^]_i_ was calculated using the formula: free [Ca^2+^]_cyt_ = K_d_ [F − F_min_]/[F_max_ − F]^33^, where K_d_ (for Fluo-4) = 345 nM. (ii) To measure ER Ca^2+^ release, cells (5 × 10^5^) stably infected with retrovirus carrying a pRS empty vector or pRS-sh*OPRM1* were loaded with 2.5 μM Mag-Fluo-4 AM for 30 min, washed with and resuspended in intracellular medium (ICM: 10 mM HEPES, pH 7.4, 19 mM NaCl, 125 mM KCl, 1 mM EGTA). ER Ca^2+^ release upon addition of increasing concentrations of d,l-methadone (0.25, 0.5 and 1 μg/ml) ± 2 μM XeC or 1 mM Tet was measured every 2 s using a Shimadzu RF 5301PC spectrofluorometer at λ_ex_ = 495_ nm_ and λ_em_ = 530_ nm_. (iii) To measure [Ca^2+^]_i_ transients by single cell Ca^2+^ imaging, cells grown in poly-L-ornithine-coated glass coverslips were loaded with 5 μM Fura-2 AM in Ca^2+^-free Krebs–Ringer–Henseleit (KRH) buffer (25 mM HEPES, pH 7.4, 125 mM NaCl, 5 mM KCl, 6 mM glucose, 1.2 mM MgCl_2_ and 2 μM EGTA), containing 0.02% Pluronic F-127 and 0.1 mg/ml BSA, for 20 min at room temperature. Coverslips were mounted in a perfusion chamber (Warner Instruments) and Ca^2+^ transients were analyzed by ratiometric single cell Ca^2+^ imaging while perfusing initially with Ca^2+^-free KRH buffer for 3 min followed by buffer containing 0.5 μg/ml d,l-methadone for 30 min then by buffer containing 5 μM TBHQ for 20 min. (iv) To measure Ca^2+^ entry and extrusion in cells loaded with 5 μM Fura-2 AM, internal Ca^2+^ stores were first vacated by treatment with 10 μM TBHQ in Ca^2+^-free/EGTA-containing KRH buffer then treated with 0.5 μg/ml d,l-methadone + 10 μM TBHQ, followed by repletion with 2 mM external Ca^2+^ to initiate Ca^2+^ entry. Where indicated, the buffer was switch to Ca^2+^-free buffer containing 200 μM EGTA and 10 μM TBHQ to stop Ca^2+^ entry. [Ca^2+^]_i_ was monitored by capturing time lapse images every 5 s using Nikon TE2000-S inverted microscope, and analysis using the Compix Simple PCI 6 software^34^. Fluorescence intensities were measured in individual cells (n = 10). Fura-2 filters have λ_ex_ = 340 ± 26 and 387 ± 11_ nm_ and λ_em_ = 510 ± 84_ nm_, and dichroic mirror (410_ nm_).

### Western blot analysis

Cell lysates were analyzed by 12.5% SDS-PAGE and immunoblotting for PARP1, caspase-3, caspase-12, Bid, calpain 1 and actin. Immunoreactive bands were detected by enhanced chemiluminescence and visualized using the Bio-Rad ChemiDoc Imager at the optimal exposure setup. Ratios of protein bands of interest vs actin were determined after densitometry using the NIH ImageJ 1.61 software.

### Analysis for apoptosis

POETIC2 cells (5 × 10^5^) stably infected with retrovirus carrying a pRS empty vector or pRS-sh*OPRM1* were seeded in 6-cm dishes, pre-treated or untreated with 0.5 μM BAPTA-AM for 30 min, and treated with 0.5 μg/ml d,l-methadone, 50 mIU l-asparaginase for 12 h. Cells were then harvested, washed twice with 1× PBS, stained with Annexin V-FITC (2 μl) and propidium iodide (2 μl), and analysed using an Attune NxT flow cytometer (ThermoFisher Scientific, USA).

### Measurement of cytosolic cytochrome C level

Cytosolic and mitochondrial fractions were isolated as described previously^35^. Briefly, cells were harvested by centrifugation at 370×*g* for 10 min, washed with 10 packed cell volumes of NKM buffer (1 mM Tris–HCl, pH 7.4, 0.13 M NaCl, 5 mM KCl and 7.5 mM MgCl_2_) and resuspended in 6 packed cell volumes of homogenization buffer (10 mM Tris–HCl, pH 6.7, 10 mM KCl, 0.15 mM MgCl_2_, 1 mM PMSF and 1 mM DTT). Cells were then homogenized using a glass homogenizer (30 strokes), resuspended in 2 M sucrose solution and centrifuged at 1200×*g* for 5 min. The supernatant was subjected to further centrifugation at 7000×*g* for 10 min and the resulting supernatant was designated as cytosolic fraction. Pellets containing mitochondria were resuspend in 3 packed cell volumes of mitochondrial suspension buffer (10 mM Tris–HCl, pH 6.7, 0.15 mM MgCl_2_, 0.25 M sucrose, 1 mM PMSF and 1 mM DTT) and centrifuged at 10,000×*g* for 5 min. Pellets were designated as mitochondrial fraction. The cytosolic and mitochondrial fractions were analyzed by SDS-PAGE and immunoblotting for cytochrome C (cyt C), tubulin and VDAC-1. Ratios of cyt C vs tubulin or VDAC1 band intensities were determined after densitometry using NIH ImageJ 1.61. Standard deviations of the calculated ratios from three independent sets of experiments were determined.

### Statistical analysis

Student’s t-test (unpaired, two-tailed) was used. Significance was set at *p* < 0.05.

## Results

### d,l-Methadone induces leukemic cell apoptosis through activation of OPRM1

**Figure 1:**
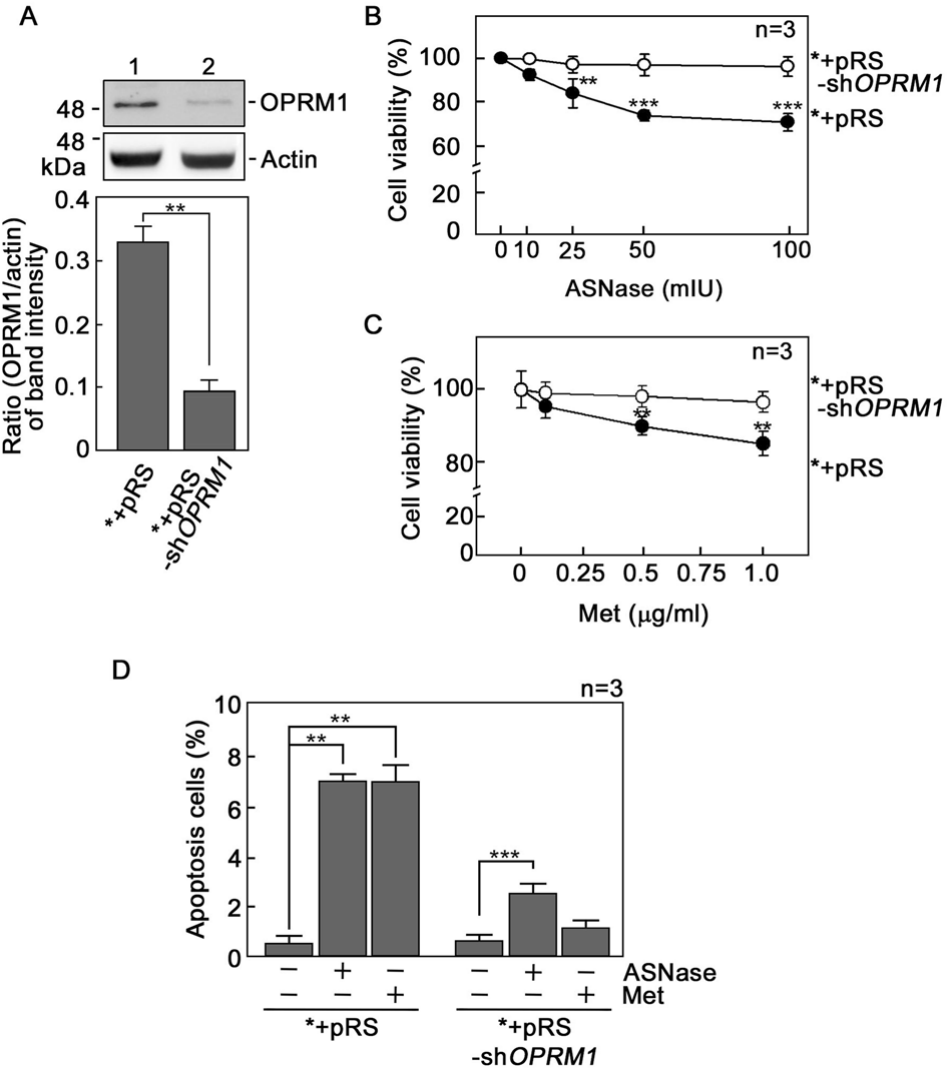
d,l-Methadone induces leukemic cell apoptosis through activation of OPRM1. (**A**) *OPRM1* depletion (upper panel) in POETIC2 leukemic cells (*) infected with retrovirus carrying pRS-sh*OPRM1* (*+pRS-sh*OPRM1*) but not in cells infected with retrovirus carrying an empty vector (*+pRS). Representative blots are from one of three independent experiments (n = 3) showing similar results. Actin blot was used as loading control. The bottom panel shows the ratios of OPRM1 vs actin levels, which were measured by densitometric analysis of the blots using NIH ImageJ 1.61. Actin levels were normalized to 1.0. Standard deviation of the OPRM1 vs actin ratio was calculated from the three sets of experiments **p < 0.0001. (**B**) and (**C**) *+pRS-sh*OPRM1* cells exhibit resistance to l-asparaginase (ASNase: **B**) and d,l-methadone (Met: **C**). Cells were treated with increasing concentrations of l-asparaginase or d,l-methadone for 24 h and surviving cells were quantified using Alamar blue assay. Values are means ± SEM from three independent experiments. **p < 0.02 and ***p < 0.001. (**D**) l-asparaginase and d,l-methadone induce apoptosis in *+pRS cells, but OPRM1 loss in *+pRS-sh*OPRM1* cells inhibits l-asparaginase- and d,l-methadone-induced apoptosis. Flow cytometry analysis was performed in cells treated with 50 mIU ASNase or 0.5 μg/ml Met for 12 h. Values are means ± SEM from three independent experiments. **p < 0.0001 and ***p < 0.033.

An opioid receptor has been implicated in d,l-methadone-induced leukemic cell apoptosis^5^, but the specific identity of this opioid receptor was not determined. Since we previously found that presence or absence of OPRM1 determines the fate of *a*LL cells following l-asparaginase treatment, i.e., presence leads to apoptosis while absence leads to survival or resistance^31^, we sought to examine the possibility that OPRM1 is targeted by d,l-methadone to induce leukemic cell apoptosis. To do so, we utilized POETIC2 cells (*) infected with retrovirus carrying a pRS empty vector (*+pRS) or pRS-sh*OPRM1* (*+pRS-sh*OPRM1*) as model systems. POETIC2 cells are continuously growing leukemia cells established from a 14-year-old patient diagnosed with pre-B *a*LL^31^. As expected, OPRM1 loss in *+pRS-sh*OPRM1* cells (Fig. 1A) inhibited l-asparaginase-induced apoptosis (Fig. 1B, D; Supplementary Figs. 1 and 2). d,l-Methadone-induced apoptosis was also inhibited by loss of OPRM1 (Fig. 1C, D; Supplementary Figs. 1 and 2), indicating that d,l-methadone induces leukemic cell apoptosis through activation of OPRM1.

**Figure 2:**
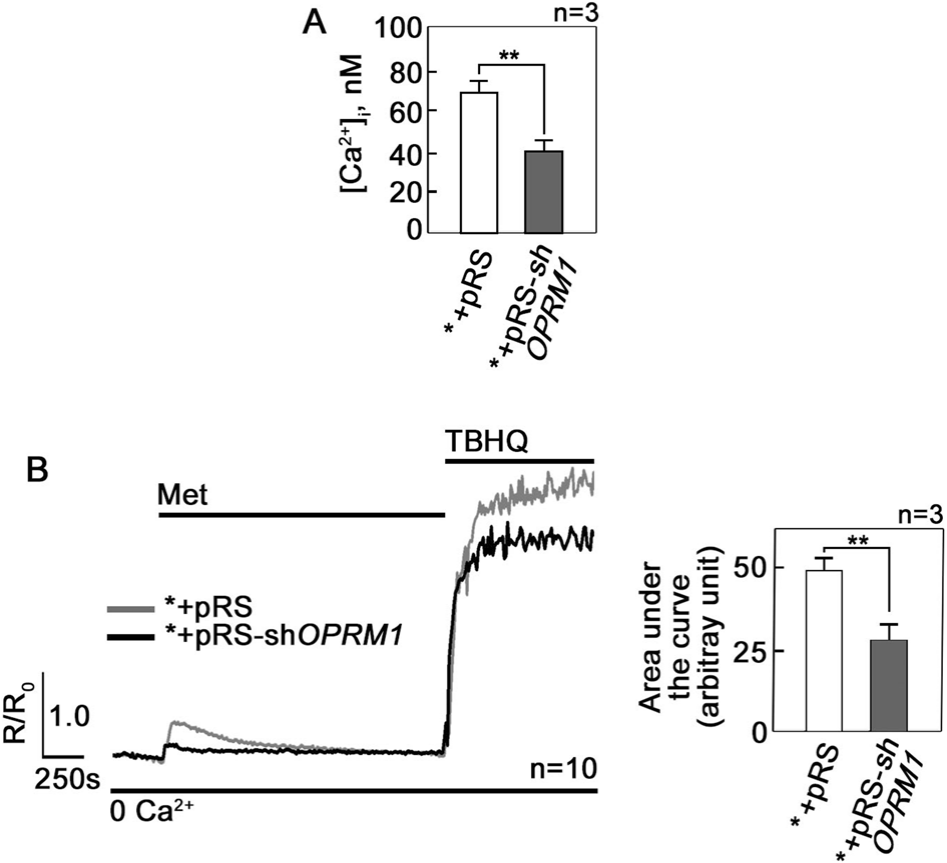
d,l-Methadone evokes Ca^2+^ release from internal stores, causing increased [Ca^2+^]_i_ that is inhibited by loss of OPRM1. (**A**) Resting [Ca^2+^]_i_ levels in *+pRS and *+pRS-sh*OPRM1* cells were measured as described in Materials and Methods. Values are means ± SEM from three independent experiments (n = 3). **p < 0.018. (**B**) Cells loaded with Fura-2 AM and treated with d,l-methadone were analyzed for Ca^2+^ transients in Ca^2+^-free buffer by ratiometric single-cell Ca^2+^ imaging using a Shimadzu RF 5301PC spectrofluorometer as described in Materials and Methods. Values represent means of Ca^2+^ signal traces in 10 cells from one of three independent experiments showing similar results (n = 3). Ca^2+^ response following TBHQ addition indicates that cells were viable during the assay. Analysis of the Ca^2+^ signal traces (left panel) indicates that d,l-methadone-induced increase in [Ca^2+^]_i_ is greater in *+pRS cells compared to *+pRS-sh*OPRM1* cells. Values are means ± SEM from three independent experiments (n = 3). **p < 0.025.

### d,l-Methadone evokes Ca^2+^release from internal stores, causing increased [Ca^2+^]_i_ that is inhibited by loss of OPRM1

d,l-Methadone was shown to induce leukemic cell death though stimulation of G_αi_, reducing [cAMP]_i_ and activating caspase-9 and -3^5^. However, in neurons, OPRM1 was implicated in intracellular Ca^2+^ homeostasis^36^, perturbation of which can cause apoptosis. Thus, we aimed to determine whether d,l-methadone-induced apoptosis in leukemic cells is associated with altered [Ca^2+^]_i_. [Ca^2+^]_i_ is regulated by release of Ca^2+^ from internal stores, influx of extracellular Ca^2+^, and extrusion of intracellular Ca^2+^. Initial analysis for resting [Ca^2+^]_i_ revealed increased level in *+pRS cells compared to OPRM1-depleted *+pRS-sh*OPRM1* cells (Fig. 2A). We then examined whether d,l-methadone alters [Ca^2+^]_i_ through deregulation of Ca^2+^ release from internal stores. To do so, cells loaded with ratiometric Fura-2 AM were maintained in Ca^2+^-free/EGTA-containing KRH buffer. By single-cell Ca^2+^ imaging, we found that d,l-methadone elicited a prompt increase in [Ca^2+^]_i_ in both *+pRS and *+pRS-sh*OPRM1* cells (Fig. 2B), suggesting that d,l-methadone stimulates Ca^2+^ release from internal stores. However, the d,l-methadone-induced rise in [Ca^2+^]_i_ was reduced in *+pRS-sh*OPRM1* cells, indicating that the d,l-methadone-induced Ca^2+^ release from internal stores occurs via the OPRM1 pathway. Treatment with TBHQ^37^, a potent inhibitor of the sarco/endoplasmic reticulum Ca^2+^ ATPase (SERCA) pump, triggered the release of internal Ca^2+^ stores, indicating that the *+pRS and *+pRS-sh*OPRM1* cells were viable during analysis.

### d,l-Methadone evokes OPRM1-mediated Ca^2+^release from the ER through the IP3R Ca^2+^ channel

**Figure 3:**
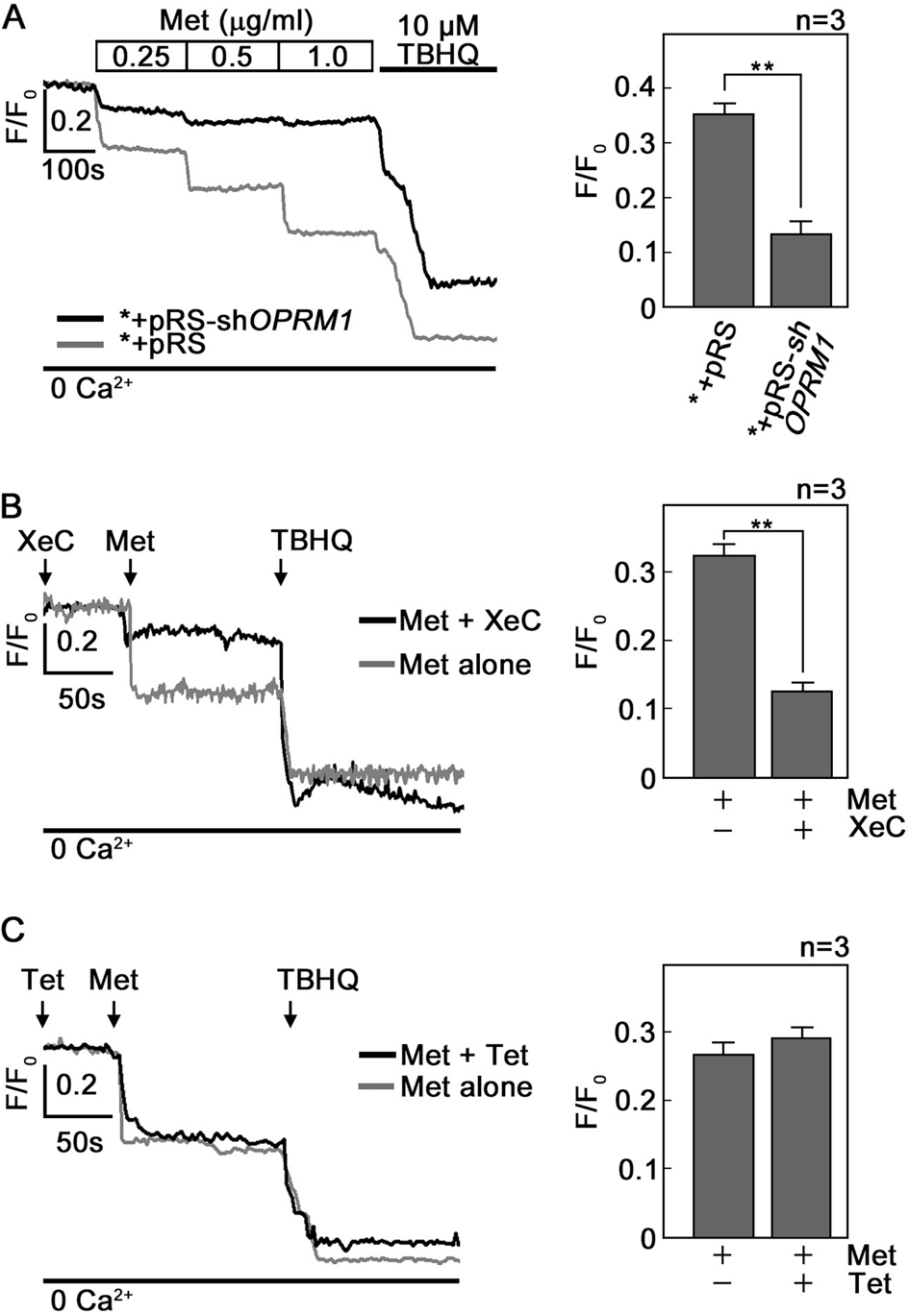
d,l-Methadone induces IP3R-mediated ER Ca^2+^ release that is inhibited by OPRM1 loss. (**A**) d,l-methadone induces ER Ca^2+^ release that is inhibited in OPRM1-depleted cells. *+pRS and *+pRS-sh*OPRM1* cells were loaded with Mag-Fluo-4 AM for 30 min then treated with increasing concentrations of d,l-methadone (0.25, 0.5 and 1 μg/ml) then analyzed for ER Ca^2+^ release by Ca^2+^ imaging. The chart on the right panel shows ER Ca^2+^ release upon treatment of cells with 0.5 μg/ml d,l-methadone. (**B**) and (**C**). d,l-Methadone-evoked ER Ca^2+^ release occurs via the IP3R channel. Thirty min after loading with Mag-Fluo-4 AM, *+pRS cells were treated with xestospongin C (XeC, in **B**) or tetracaine (Tet in **C**) for 10 min. ER Ca^2+^ release upon d,l-methadone addition was then measured every 2 s. The right panels show ER Ca^2+^ release upon treatment with d,l-methadone with or without prior treatment with XeC (**B**) or Tet (**C**). Representative data from one of three independent experiments (n = 3) showing similar results. Ca^2+^ response after TBHQ addition indicates that cells were viable during the assay. Values are means ± SEM from three independent experiments (n = 3). **p < 0.02.

We next sought to determine whether the d,l-methadone-induced rise in [Ca^2+^]_i_ in leukemic cells is due to Ca^2+^ release from the ER. To do so, cells loaded with an ER Ca^2+^ probe^38^, Mag-Fluo-4 AM, then treated with d,l-methadone were analyzed for ER Ca^2+^ release by spectrofluorometric Ca^2+^ imaging. As shown in Fig. 3A, d,l-methadone induced Ca^2+^ release from the ER but *+pRS-sh*OPRM1* cells showed reduced ER Ca^2+^ release compared to *+pRS cells, indicating inhibition of Ca^2+^ release by loss of OPRM1 and, therefore, d,l-methadone-induced ER Ca^2+^ release is mediated by OPRM1. We then tested whether d,l-methadone-induced ER Ca^2+^ release occurs through the IP3R and/or the ryanodine receptor (RyR), both of which form Ca^2+^ channels in the ER^39^. For this experiment, Mag-Fluo-4 AM-loaded and d,l-methadone-stimulated cells were treated with xestospongin C (XeC, a potent IP3R inhibitor: Fig. 3B)^40^ or tetracaine^41^ (Tet, a potent RyR inhibitor: Fig. 3C) and analyzed for ER Ca^2+^ release. As shown in Fig. 3, XeC (Fig. 3B) but not Tet (Fig. 3C) inhibited d,l-methadone-induced ER Ca^2+^ release, indicating that such Ca^2+^ release is mediated by IP3R and not by RyR.

### The rate of Ca^2+^ extrusion following d,l-methadone treatment is slower in *+pRS cells compared to *+pRS-sh*OPRM1* cells

**Figure 4:**
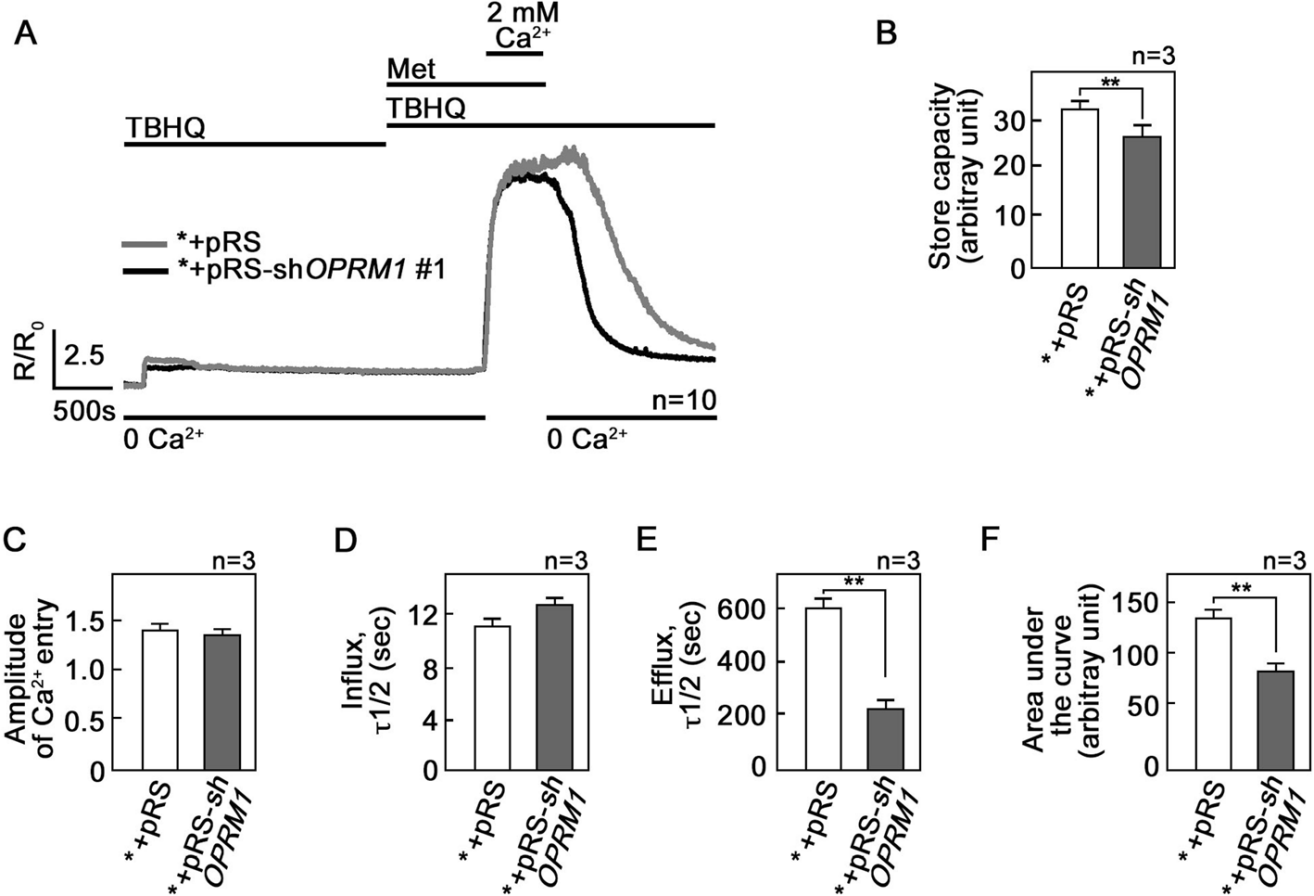
The rate of Ca^2+^ extrusion following d,l-methadone treatment in leukemic cells is accelerated by loss of OPRM1. (**A**) External Ca^2+^ entry and intracellular Ca^2+^ extrusion in *+pRS and *+pRS-sh*OPRM1* cells following d,l-methadone treatment. [Ca^2+^]_i_ was measured in cells loaded with Fura-2 AM and treated with TBHQ with or without d,l-methadone or external Ca^2+^ using single-cell Ca^2+^ imaging. Values represent means of Ca^2+^ signal traces from 10 cells. (**B**) Internal Ca^2+^ store capacity in OPRM1-depleted *+pRS-sh*OPRM1* cells is reduced compared to that in control *+pRS cells. Release of internal Ca^2+^ stores upon treatment with TBHQ allows measurement of internal Ca^2+^ store capacity. In the presence of d,l-methadone, there is no difference in (**C**) the amplitude of Ca^2+^ entry and (**D**) the rate of [Ca^2+^]_i_ influx in *+pRS and *+pRS-sh*OPRM1* cells. (**E**) The rate of Ca^2+^ extrusion, represented by the decline in [Ca^2+^]_i_ following removal of external Ca^2+^, is faster (i.e., reduced T_1/2_) in *+pRS-sh*OPRM1* cells than in *+pRS cells. (**F**) Comparison of the integrated Ca^2+^ signals [area under the curve from the start of the Ca^2+^ signal (at 50 min) until 25 min later (at 75 min)] shows decreased Ca^2+^ transient in *+ pRS-sh*OPRM1* cells compared to *+pRS cells. Values in (B)–(F) are means ± SEM from three independent experiments (n = 3). **p < 0.025.

Our next step was to explore the possibility that OPRM1 loss also affects extracellular Ca^2+^ influx and intracellular Ca^2+^ extrusion following treatment with d,l-methadone. To do so, internal Ca^2+^ stores in Fura-2 AM-loaded cells were initially emptied by treatment with TBHQ in Ca^2+^-free/EGTA-containing buffer. Cells were then treated with d,l-methadone in the presence of TBHQ followed by 2 mM (Fig. 4A) or 500 mM Ca^2+^ (Supplementary Fig. 3), which was added to the external buffer to initiate Ca^2+^ entry. Ca^2+^ extrusion was also measured in the presence of TBHQ following a switch to Ca^2+^-free/EGTA-containing buffer. The initial treatment with TBHQ, which causes the release of internal Ca^2+^ stores, allows measurement of internal Ca^2+^ store capacity, and as shown in Fig. 4A, B, OPRM1-depleted *+pRS-sh*OPRM1* cells have reduced capacity compared to *+pRS cells. In the presence of d,l-methadone and upon external Ca^2+^ addition, there was no difference in Ca^2+^ entry between *+pRS and *+pRS-sh*OPRM1* cells (Fig. 4C) as well as in their rates of Ca^2+^ influx as measured by T_1/2_ of influx (Fig. 4D). However, upon switching to Ca^2+^-free/EGTA-containing buffer and withdrawal of d,l-methadone but continued presence of TBHQ, the rate of Ca^2+^ extrusion was faster (i.e., reduced T_1/2_ of efflux) in *+pRS-sh*OPRM1* cells compared to *+pRS cells (Fig. 4E), indicating that OPRM1 regulates the rate of Ca^2+^ extrusion. Thus, the integrated Ca^2+^ signals, which correspond to the calculated area under the curve [i.e., from the beginning to the end (back to baseline) of Ca^2+^ signal] was reduced in OPRM1-deficient *+pRS-sh*OPRM1* cells compared to control *+pRS cells (Fig. 4F).

### d,l-Methadone-induced leukemic cell apoptosis is linked to increased [Ca^2+^]_i_

**Figure 5:**
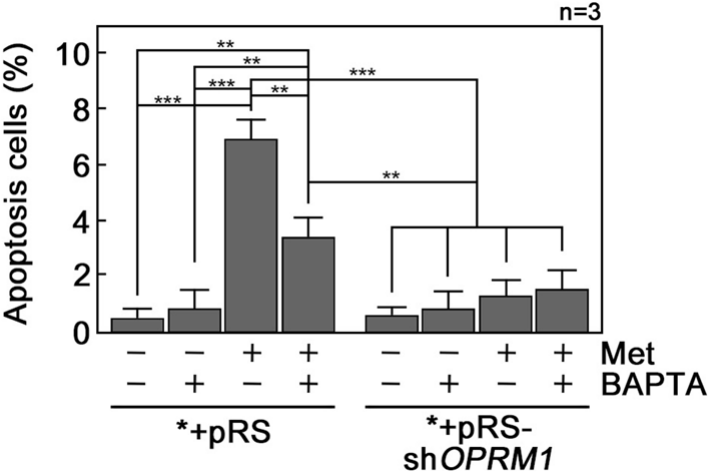
d,l-Methadone-induced apoptosis is reversed by Ca^2+^ chelation with BAPTA-AM. *+pRS and *+pRS-sh*OPRM1* cells pre-treated or untreated with 50 μM BAPTA-AM (BAPTA) for 30 min then treated or untreated with 0.5 μg/ml d,l-methadone for 12 h were stained with PI and FITC-Annexin V and subjected to flow cytometry as described in Materials and Methods. Values are means ± SEM from three independent experiments (n = 3). **p < 0.01 and ***p < 0.025.

Since we found that d,l-methadone, which causes leukemic cell apoptosis, triggers IP3R-mediated ER Ca^2+^ release and delays the rate of Ca^2+^ extrusion, causing increased [Ca^2+^]_i_, we next wished to establish a link between the d,l-methadone-associated rise in [Ca^2+^]_i_ and d,l-methadone-induced apoptosis. Leukemic cells were treated with d,l-methadone in the presence or absence of the Ca^2+^ chelator, BAPTA-AM, then subjected to flow cytometry analysis after staining with PI and FITC-Annexin V. As shown in Fig. 5, d,l-methadone induced *+pRS cell apoptosis that was inhibited by BAPTA-AM, indicating that d,l-methadone-induced apoptosis is linked to increased [Ca^2+^]_i_. Consistent with our results above, loss of OPRM1 in *+pRS-sh*OPRM1* cells inhibited d,l-methadone-induced apoptosis, which was unaffected by BAPTA-AM.

### d,l-methadone induces leukemic cell apoptosis by upregulating the Ca^2+^-mediated calpain-1-Bid-cytochrome C-caspase-3/12 apoptotic pathway

**Figure 6:**
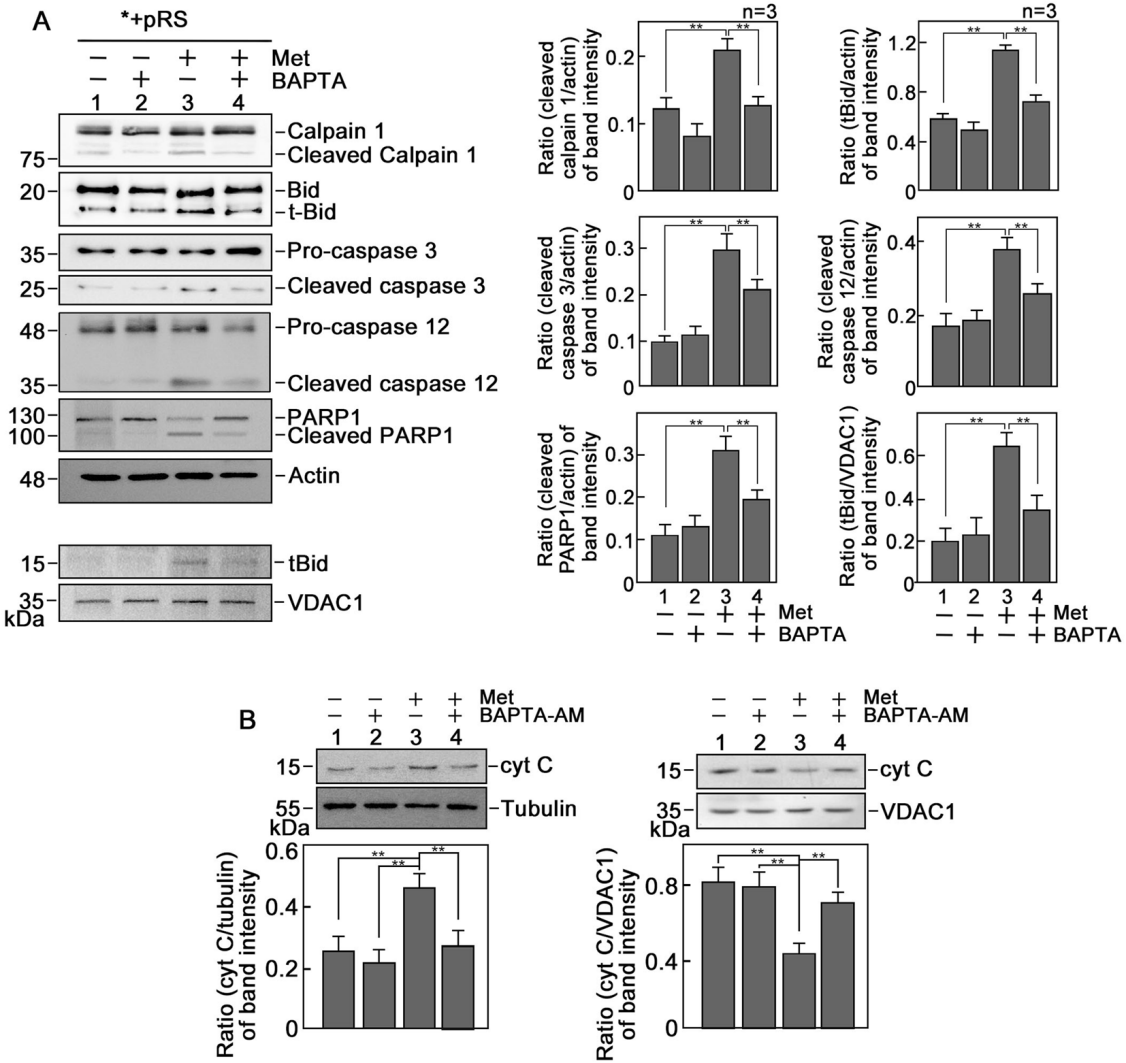
d,l-Methadone induces apoptosis through upregulation of the Ca^2+^-mediated calpain-1-Bid-cytochrome C-caspase-3/12 apoptotic pathway. (**A**) Lysates of cells treated with d,l-methadone in the presence or absence of BAPTA-AM for 24 h were analyzed by SDS-PAGE and immunoblotting for calpain 1, Bid, caspase-3, cleaved caspase-3, caspase-12, PARP1 and actin. Actin blot was used as loading control. Mitochondrial fractions from cells treated as described above were also analyzed by SDS-PAGE and immunoblotting for t-Bid and VDAC1. Representative blots are from one of three independent experiments (n = 3) showing similar results. Charts on the right panel show the ratios of the levels of apoptosis-associated proteins vs actin or VDAC1, with the actin and VDAC1 values normalized to 1.0. The charts correspond to the densitometry analysis of the representative blots shown on the left panel. (**B**) d,l-Methadone increases cytochrome C (cyt C) level in the cytosol. Cytosolic and mitochondrial fractions from cells pre-treated or untreated with 0.5 μM BAPTA-AM for 30 min then treated or untreated with 0.5 μg/ml d,l-methadone for 12 h were analyzed by SDS-PAGE and immunoblotting for cyt C, tubulin and VDAC1 (upper panels). Tubulin and VDAC1 were used as loading controls for cytosolic and mitochondrial fractions, respectively. Representative blots are from one of three independent experiments showing similar results. The lower panels show the ratios of cyt C vs tubulin or VDAC1 levels, with tubulin or VDAC1 levels normalized to 1.0. Values are means ± SEM of the three independent experiments (n = 3). **p < 0.05.

Previously, d,l-methadone-induced leukemic cell apoptosis was linked to activation of caspase-9 and -3 as well as downregulation of BCL^6^. zVAD.fmk, the broad-spectrum inhibitor of caspases, almost completely inhibits d,l-methadone-induced leukemic cell apoptosis, indicating that caspases (together with BCL activation) is the main route for apoptosis. Here, we sought to analyze the expression of some of the components of the Ca^2+^-mediated apoptotic pathway in *+pRS leukemic cells treated with d,l-methadone. As shown in Fig. 6A, levels of activated calpain-1, t-Bid, caspase-3 and caspase-12 were elevated in d,l-methadone-treated *+pRS cells, and these were reversed by treatment with BAPTA-AM. A similar pattern was observed in the caspase-3-mediated PARP1 cleavage product. Inhibition of calpain-1 by calpeptin reduced d,l-methadone-induced leukemic cell apoptosis (Supplementary Fig. 4), indicating the involvement of calpain-1 in d,l-methadone-induced leukemic cell apoptosis. As expected, translocation of t-Bid from the cytosol to mitochondria was observed (Fig. 6A, left and right bottom panels). Since activation of these elements of the Ca^2+^-mediated apoptotic pathway induces mitochondrial cytochrome c (cyt C) release into the cytosol, which leads to the activation of downstream caspases and subsequent apoptosis^42,43^, we also examined cytosolic and mitochondrial cyt C levels in *+pRS cells following treatment with d,l-methadone. As shown in Fig. 6B, d,l-methadone caused an increase in cyt C level in the cytosol and corresponding decrease in mitochondria, which was reversed by BAPTA-AM treatment. We also assessed mPTP opening in d,l-methadone-induced leukemic cell apoptosis by calcein-AM staining followed by treatment with CoCl_2_. Calcein-AM is a cell permeable fluorophore that diffuses and gets trapped in all subcellular compartments, including mitochondria. Treatment with cobalt (Co^2+^) quenches calcein fluorescence in all subcellular compartments except the mitochondrial matrix which is enclosed by a Co^2+^ impermeable inner mitochondrial membrane when mPTP is closed. Thus, the ability of Co^2+^ to quench mitochondrial calcein fluorescence only when mPTP is open allows determination of open vs closed status of mPTP in the cell. Upon treatment with CoCl_2_, d,l-methadone treatment did not alter calcein fluorescence intensity, indicating that increased mPTP opening is not involved in d,l-methadone-induced apoptosis (Supplementary Fig. 5). Altogether, our findings support our view that d,l-methadone induces OPRM1-regulated apoptosis by increasing IP3R-mediated ER Ca^2+^ release and slowing down intracellular Ca^2+^ extrusion, causing a rise in [Ca^2+^]_i_ and upregulating the calpain-1-Bid-cytochrome C-caspase-3/12 apoptotic pathway.

## Discussion

**Figure 7:**
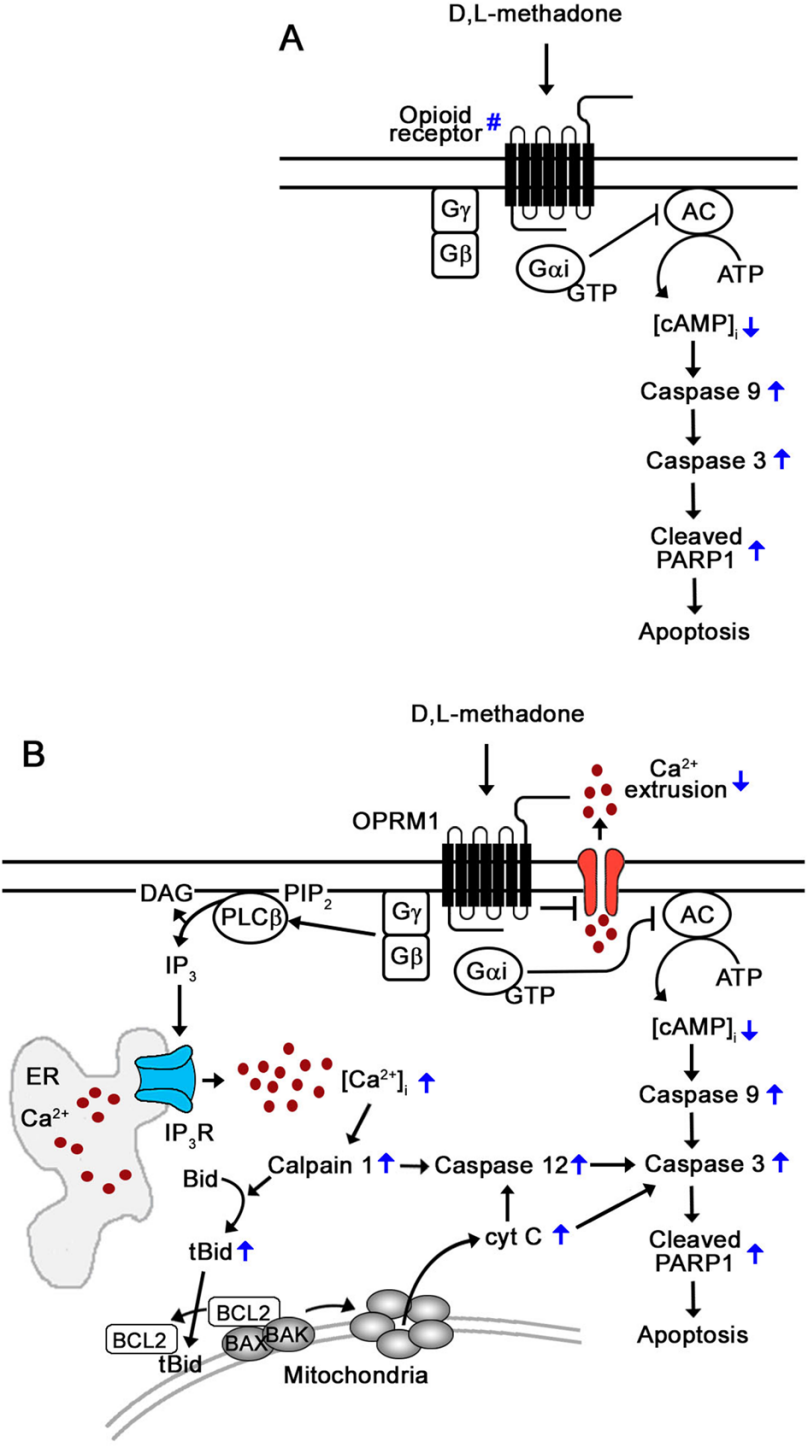
Proposed mechanism for OPRM1-mediated d,l-methadone-induced apoptosis in leukemic cells. (**A**) Previously, stimulation of an unidentified type of opioid receptor (#) by d,l-methadone was shown to induce leukemic cell death via G_αi_, which blocks adenylyl cyclase activity that in turn reduces [cAMP]_i_, activating caspase-9 and -3^5^. (**B**) In the current study, we identify the OPRM1 opioid receptor as a specific d,l-methadone target in leukemic cells. Since activation of opioid receptors has been shown to cause G_βγ_-mediated rise in [Ca^2+^]_i_ via PLC^22,26–28^, we propose that d,l-methadone activation of OPRM1 in leukemic cells causes G_βγ_-mediated stimulation of PLC, which then triggers a rise in [Ca^2+^]_i_ through increased IP3R-mediated ER Ca^2+^ release and reduced rate of Ca^2+^ efflux, causing upregulation of the Ca^2+^-mediated calpain-1-Bid-cyt C-caspase-3/12 apoptotic pathway and subsequent apoptosis.

d,l-Methadone has recently been implicated in leukemic cell apoptosis^4–6^ and this pro-apoptotic effect has been linked to the opioid receptor signaling pathway^5^. In particular, d,l-methadone was found to induce apoptosis in HL-60 human myeloid leukemia and CEM human T-cell lymphoblastic leukemia cell lines via downregulation of both X chromosome-linked inhibitor of apoptosis (XIAP) and B-cell lymphoma-extra large (Bcl‐x_L_)^4^. In xenograft-derived *a*LL cells, it was reported that d,l-methadone induces apoptosis through activation of opioid GPCRs and subsequent downregulation of cAMP and activation of caspase-9 and -3^5^ (Fig. 7A). This was deduced from the observed expression of opioid receptors in leukemic cells and the inhibition of d,l-methadone-induced apoptosis by 3-isobutyl-1-methylxanthine (IBMX), PTX, and naloxone. IBMX is a cAMP phosphodiesterase inhibitor that causes cAMP upregulation; PTX inhibits the inhibitory α subunit (G_αi_) of heterotrimeric G_αβγ_-proteins; and naloxone blocks opioid receptor activation. The indirect approach to ascertain opioid receptors as targets for d,l-methadone may, however, be questioned as the use of opioid receptor expression may be seen as a weak supporting evidence, and the use of naloxone appears to be challenged by reports that naloxone also inhibits toll-like receptor 4 (TLR4) signaling, a prominent regulator of immune cell function^44^.

The current study utilized a stringent knockdown system to evaluate and identify OPRM1 as a specific d,l-methadone target that mediates apoptosis in leukemic cells. While it is believed that opioid receptors signal through G_*α*i/o_ subunits to inhibit adenylyl cyclase activity, reducing cAMP production and protein kinase A activity^22^, opioids elicit IP3R-mediated Ca^2+^ release from the ER via PLC^45,46^. In leukocytes^22,27^, pituitary^28^ and neuroblastoma cells^26^, G_βγ_ subunit activation of PLCβ and stimulation of IP3R are associated with opioid-induced increase in [Ca^2+^]_i_. Similarly, mu-opioid receptor-mediated PLC activation through a PTX-sensitive G protein and possible IP3-mediated increase in [Ca^2+^]_i_ in neuroblastoma cells were presumed to be involved in opioid action^29^. These findings together with our previous discovery that *a*LL cell expression of OPRM1 leads to apoptosis following l-asparaginase treatment while lack of OPRM1 expression leads to survival or resistance to l-asparaginase^31^, prompted us to investigate the possibility that OPRM1 is targeted by d,l-methadone to induce leukemic cell apoptosis, and that OPRM1 regulation of [Ca^2+^]_i_ is a critical component of d,l-methadone-induced apoptotic pathway in leukemic cells.

Our studies revealed that resting [Ca^2+^]_i_ in control *+pRS leukemic cells is increased compared to OPRM1-depleted *+pRS-sh*OPRM1* cells. We established that increased [Ca^2+^]_i_ in *+pRS cells is linked to the stimulation of apoptosis by d,l-methadone, and that the rise in [Ca^2+^]_i_ is due to increased IP3R-mediated ER Ca^2+^ release and reduced rate of intracellular Ca^2+^ extrusion. These conclusions were based on the analysis of cells treated with the Mag-Fluo-4 AM Ca^2+^ probe^38^, which distinguishes the release of Ca^2+^ from the ER, and with XeC, which inhibits Ca^2+^ release via the IP3R Ca^2+^ channel^40^, as well as by introduction and withdrawal of extracellular Ca^2+^ in the presence of TBHQ, which empties internal Ca^2+^ stores^37^, allowing measurement of intracellular Ca^2+^ extrusion. These observed Ca^2+^ dynamics in *+pRS cells following treatment with d,l-methadone are reversed by loss of OPRM1. Apparently, while d,l-methadone induces ER Ca^2+^ release and delays Ca^2+^ efflux in *+pRS cells, causing a lethal rise in [Ca^2+^]_i_, loss of OPRM1 in *+pRS-sh*OPRM1* cells inhibits ER Ca^2+^ release and accelerates Ca^2+^ efflux following d,l-methadone treatment, preventing a lethal rise in [Ca^2+^]_i_, and subsequently inhibiting d,l-methadone-induced apoptosis. Thus, our findings support an OPRM1-regulated d,l-methadone-induced [Ca^2+^]_i_-mediated apoptotic pathway in leukemic cells.

In fact, we demonstrate that OPRM1-regulated Ca^2+^-mediated apoptosis induced by d,l-methadone in leukemic cells is triggered by activation of the Ca^2+^-dependent calpain-1 and associated proteolytic events mediated by cysteine proteases. Our findings are consistent with the model illustrated in Fig. 7B where Ca^2+^-activated calpain-1 targets Bid to generate its truncated form, t-Bid, which translocates to mitochondria where it binds and neutralizes the anti-apoptotic Bcl-2. This allows BAK and BAX to form pores in the outer mitochondrial membrane, causing cyt C release, activation of caspases such as caspase-12^47^ and caspase-3^48^, and subsequent apoptosis^42,43,49^. Caspase-3 cleaves PARP1, which drives apoptosis in leukemic cells^50^. Our analysis show that d,l-methadone triggers OPRM1-regulated apoptosis by inducing cleavage of calpain-1, Bid, procaspase-3, procaspase-12 and PARP1, indicating an [Ca^2+^]_i_-mediated apoptosis. This was substantiated by the reversal of apoptosis by the Ca^2+^ chelator, BAPTA-AM.

In summary, we identify OPRM1 as a novel and specific d,l-methadone target in leukemic cells. Our observations indicate that d,l-methadone activates OPRM1, which triggers an increase in IP3R-mediated ER Ca^2+^ release and decrease in the rate of Ca^2+^ efflux, causing a rise in [Ca^2+^]_i_ that upregulates the Ca^2+^-mediated calpain-1-Bid-cyt C-caspase-3/12 apoptotic pathway. We note that although loss of OPRM1 in *+pRS-sh*OPRM1* cells almost completely inhibited d,l-methadone-induced apoptosis, indicating that d,l-methadone primarily targets OPRM1 to induce leukemic cell death, the fact that BAPTA-AM only partially inhibits d,l-methadone-induced *+pRS cell apoptosis suggests the co-existence of at least one other branch of the OPRM1-mediated d,l-methadone-induced apoptotic pathway. While still under our separate investigation, we presume that the previously reported d,l-methadone-induced apoptotic pathway that downregulates cAMP is in fact a branch of the OPRM1 apoptotic pathway. Certainly, the mode of action of d,l-methadone requires further investigation if it is to be clinically deliberated as a relevant adjuvant or sensitizer for *a*LL chemotherapy.

## Supplementary Information

Supplementary Information.
