# Supplementary material for: d,l-Methadone causes leukemic cell apoptosis via an OPRM1-triggered increase in IP3R-mediated ER Ca2+ release and decrease in Ca2+ efflux, elevating [Ca2+]i
Source: Sci Rep. 2021 Jan 13;11:1009. doi: 10.1038/s41598-020-80520-w (PMC7806773; doi:10.1038/s41598-020-80520-w)

D,L-methadone causes leukemic cell apoptosis via an OPRM1-triggered increase in IP3R-mediated ER Ca^{2+} release and decrease in Ca^{2+} efflux, elevating $[Ca^{2+}]_i$

JungKwon Lee[1, 2], Jesusa L. Rosales[1], Hee-Guk Byun[2] and Ki-Young Lee[1*]

Supplementary Materials and Methods

mPTP opening assay Calcein fluorescence was measured using the Image-IT live mitochondria permeability transition pore assay kit (Thermo Fisher Sci.) as per the manufacturer's protocol. Briefly, cells (1×10^4) pre-incubated with cyclosporin A (CsA; 1 µM) for 1 hr then treated with D,L-methadone for 16 hrs were incubated with or without ionomycin (1 µM). Cells were then stained with calcein AM (1 µM) for 15 min then with $CoCl_2$ (1 mM) in Hank's balanced salt solution (HBSS) for 15 min. Cells were washed with HBSS, resuspended in ice-cold PBS and analyzed by flow cytometry using a fluorescein isothiocyanate filter (530 nm) for measuring calcein fluorescence.

Supplementary Figure Legend

Supplementary Figure 1. *+pRS-sh*OPRM1* cells exhibit resistance to L-asparaginase (ASNase) and D,L-methadone (Met). Cells were treated with 50 mIU of ASNase (left panel) or 0.5 µg/ml Met (right panel) for 24, 48, and 72 hrs. Surviving cell fractions were quantified using Alamar blue assay. Values are means ± SEM from three independent experiments. **$p<0.05$.

Supplementary Figure 2. OPRM1 loss inhibits L-asparaginase- and D,L-methadone-induced apoptosis. Flow cytometry analysis was performed in cells treated with 50 mIU ASNase or 0.5 µg/ml Met for 12 hrs. Data are presented in pseudocolor plots, with green and brown dots correspond to live and apoptotic cells, respectively. Dataset represent one of three experiments presented in Figure 1D.

Supplementary Figure 3. The pattern of Ca^{2+} extrusion following D,L-methadone treatment in the presence of 500 µM external Ca^{2+} is similar to that of 2 mM (as shown in Figure 4A) except that amplitudes of Ca^{2+} entry in both OPRM1-depleted and control cells are reduced.

Supplementary Figure 4. Activation of calpain-1 is involved in D,L-methadone-induced leukemic cell apoptosis. Cells pretreated (or untreated) with 10 µM calpeptin then treated with D,L-methadone for 16 hrs were analyzed by flow cytometry following Annexin V-FITC and propidium iodide staining. Values are means ± SEM from three (n = 3) independent experiments. **$p<0.05$.

Supplementary Figure 5. mPTP opening is not involved in D,L-methadone-induced leukemic cell apoptosis. Cells (1×10^4) treated with D,L-methadone were stained with 1 µM calcein-AM then $CoCl_2$ and analyzed by flow cytometry (left panel). 1 µM ionomycin and 1 µM cyclosporin A (CsA) were used as positive and negative controls, respectively. Quantitative analysis of the relative calcein fluorescence is shown on the right panel. Values for cells loaded with calcein-AM alone were normalized to 1.0. The relative calcein fluorescence intensity in cells treated with $CoCl_2$ were then calculated. Values are means ± SEM from three (n = 3) independent experiments. **$p<0.05$ by unpaired Student's t test.

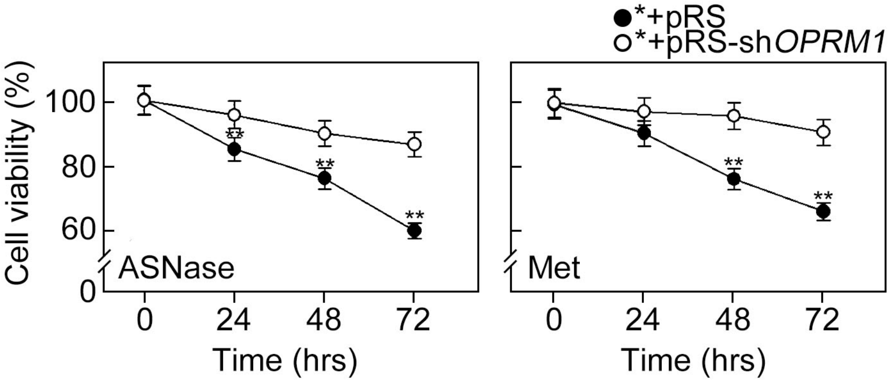

Supplementary Fig. 1

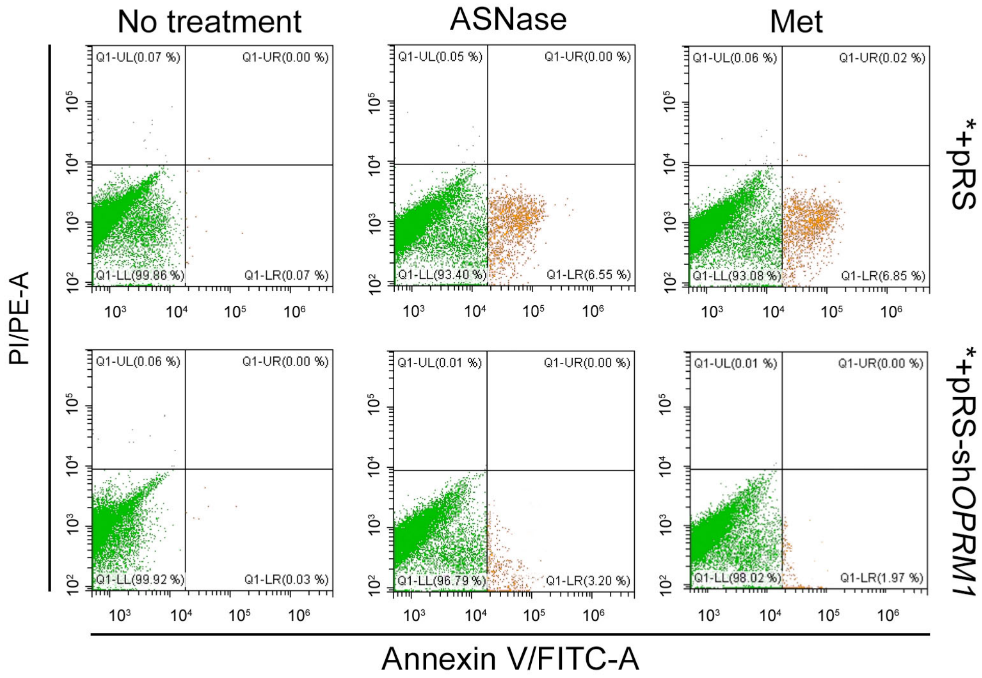

Supplementary Fig. 2

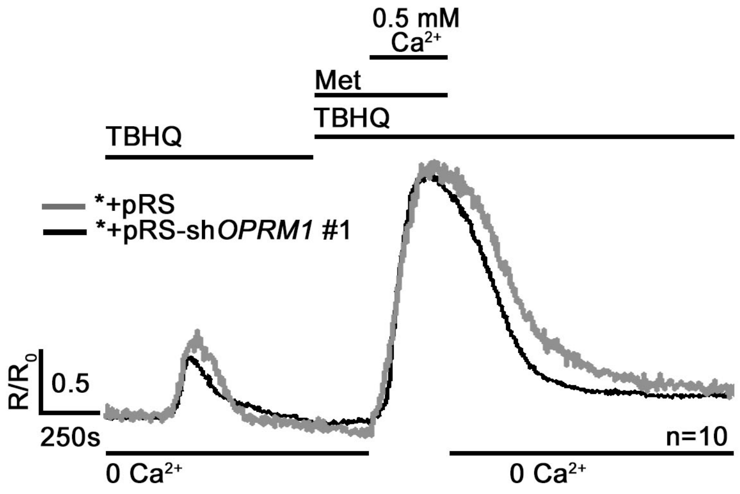

Supplementary Figure 3

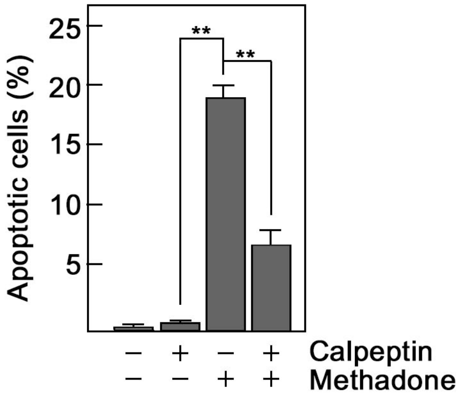

Supplementary Fig. 4

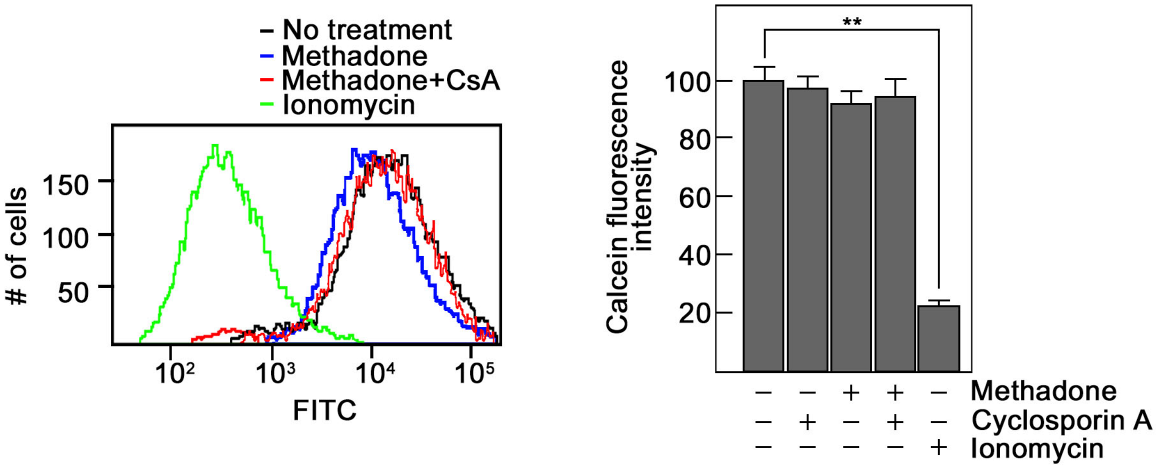

Supplementary Figure 5

Note: some of the blots were cut to allow immunoblotting with different antibodies without reblotting the whole membrane.

Figure 1A

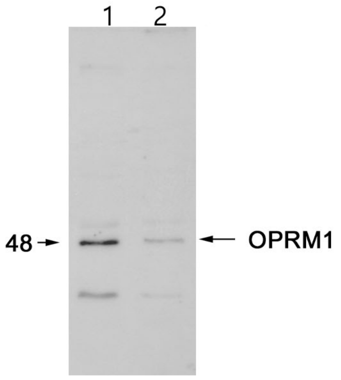

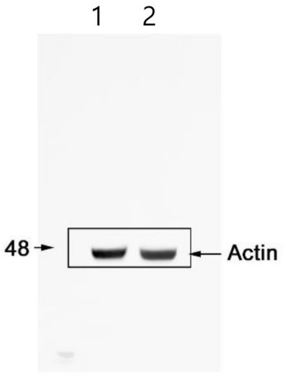

A, upper panel A, upper panel

~~Figure 1~~.

Figure 6A

Upper panel

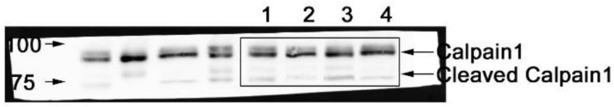

We couldn't locate upper and lower portion of the blots.

2nd panel from the top

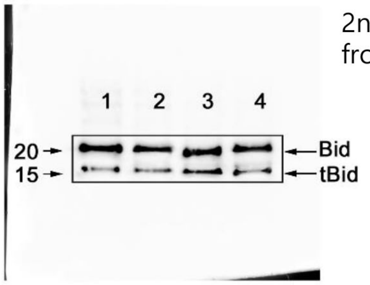

3rd and 4th panels

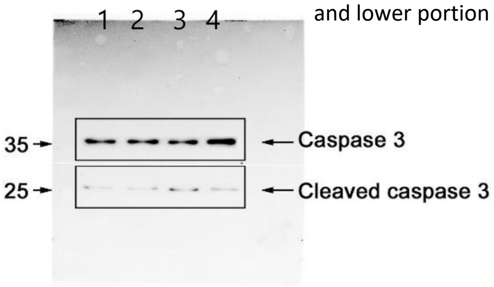

5th panel

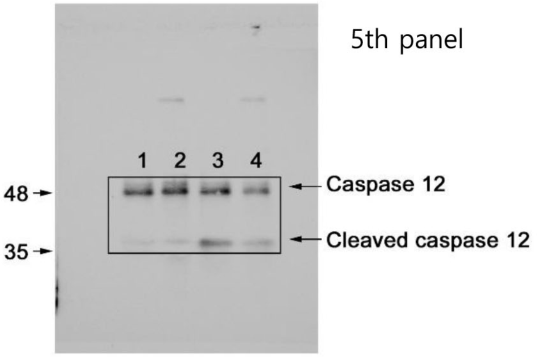

6th panel

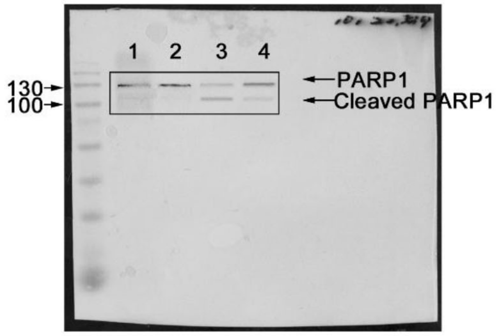

7th panel

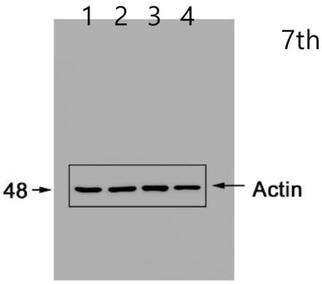

Bottom panel

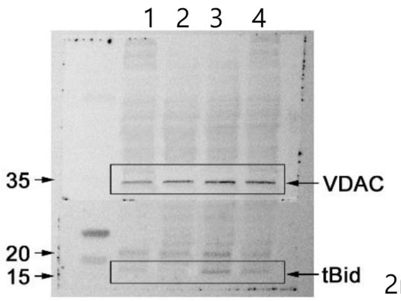

2nd to the bottom panel

~~Figure 6A~~.

Figure 6B

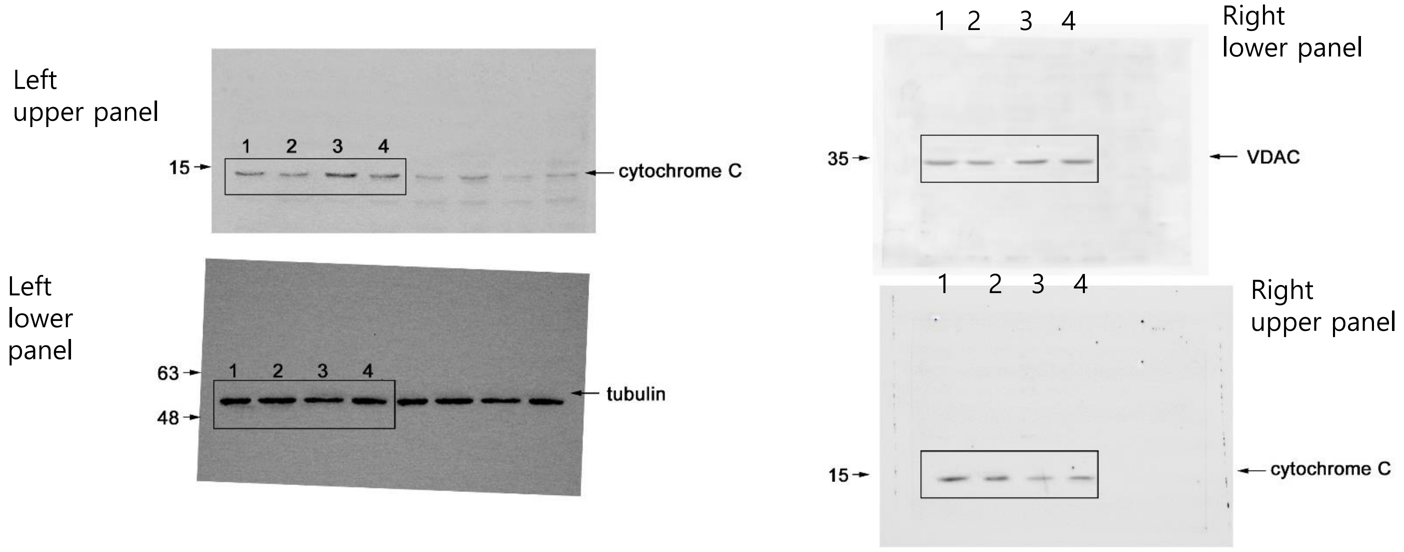

~~Figure 6B~~.

Fig. 1A additional data

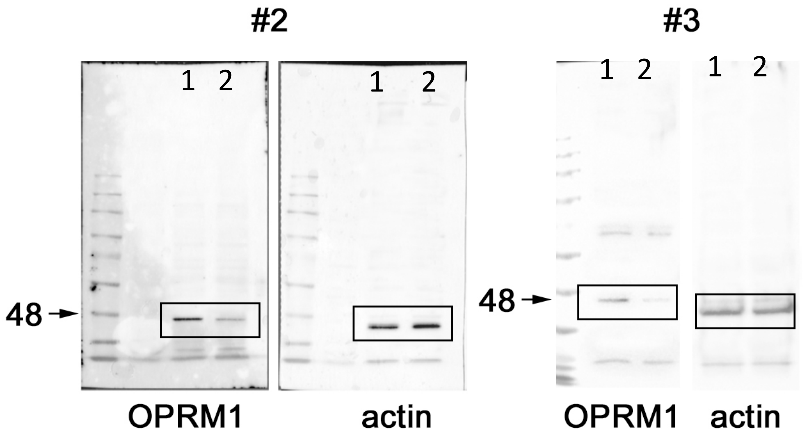

Fig. 6A additional data

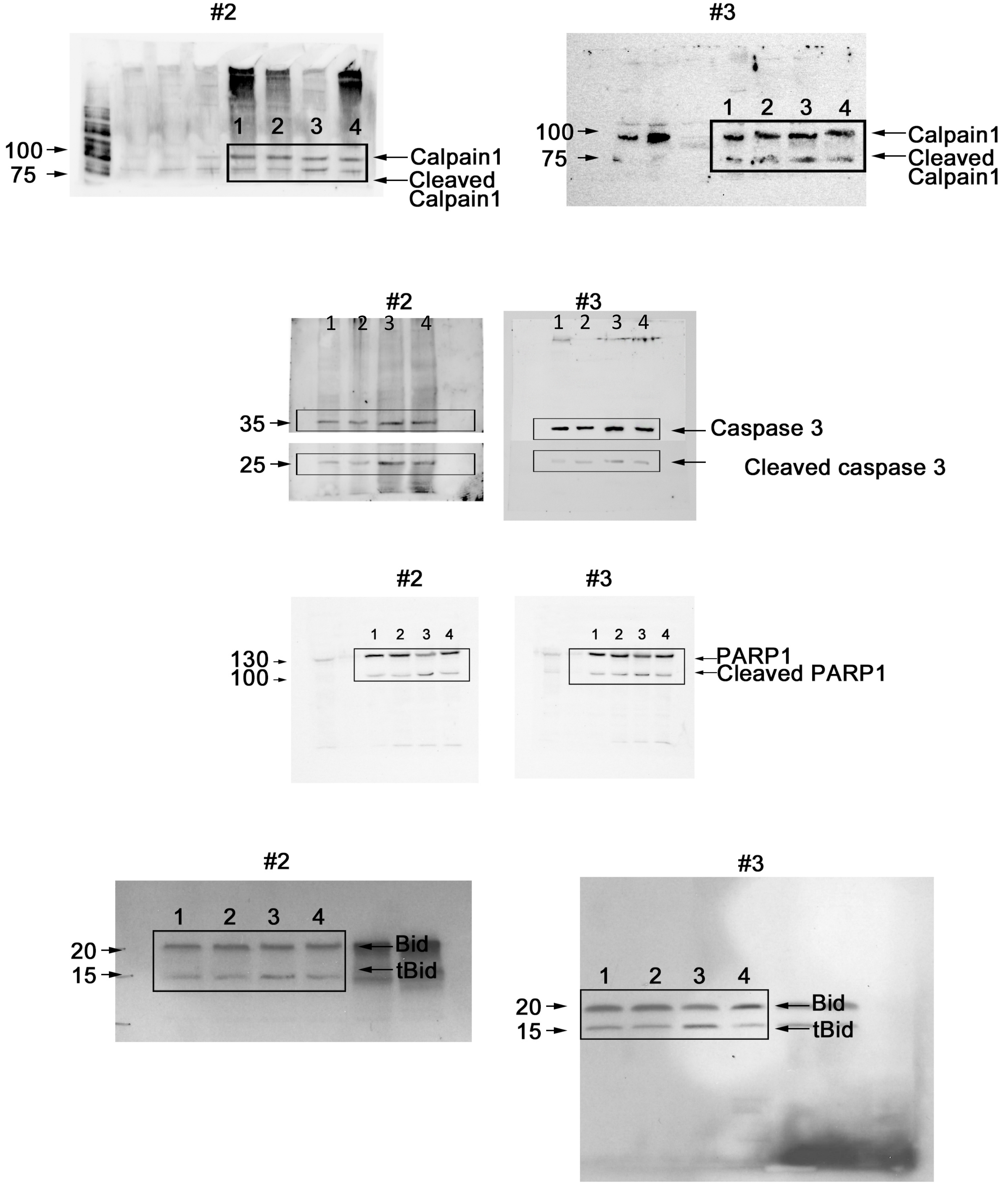

#2

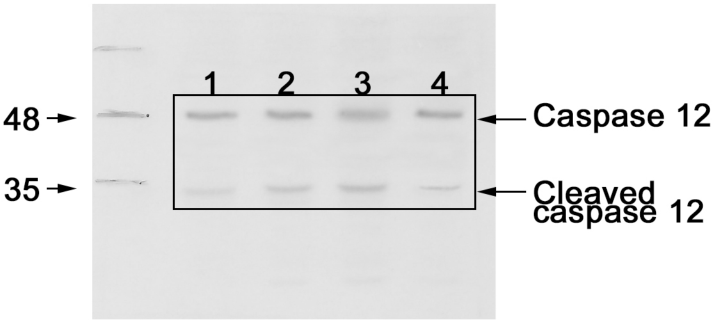
1
2
3
4
48
35
Caspase 12
Cleaved caspase 12

#3

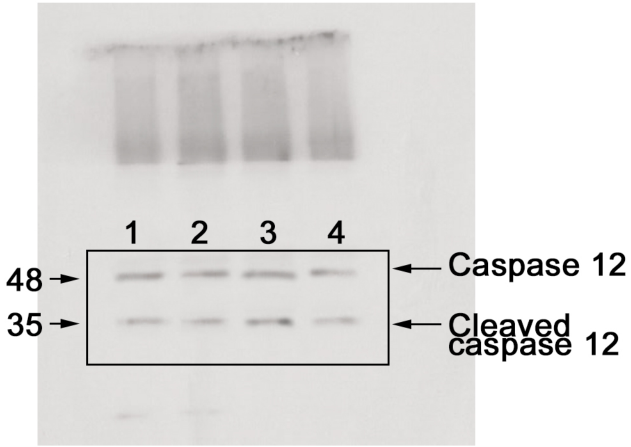
1
2
3
4
48
35
Caspase 12
Cleaved caspase 12

#2

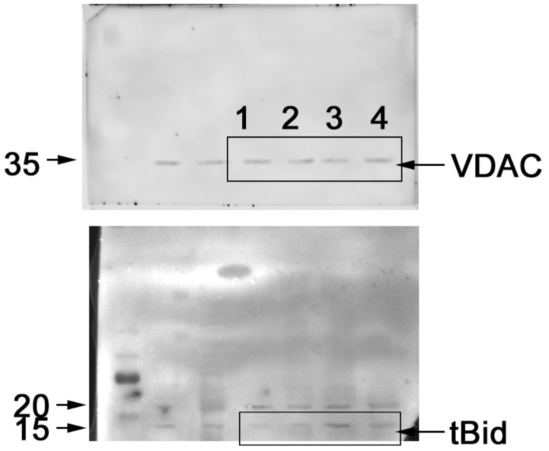
1
2
3
4
35
VDAC
20
15
tBid

#3

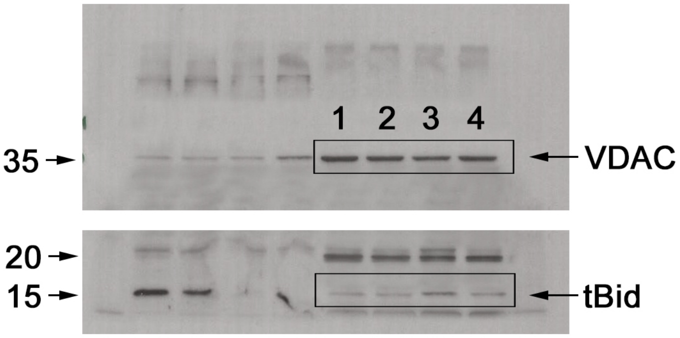
1
2
3
4
35
VDAC
20
15
tBid

#2

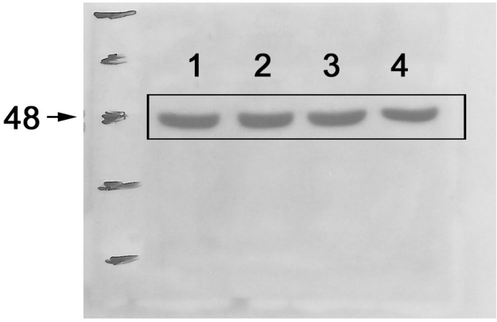
1
2
3
4
48

#3

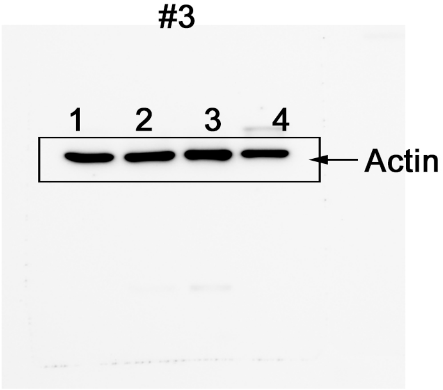
1
2
3
4
Actin

Fig. 6B additional data

For left panel

#2 **#3**

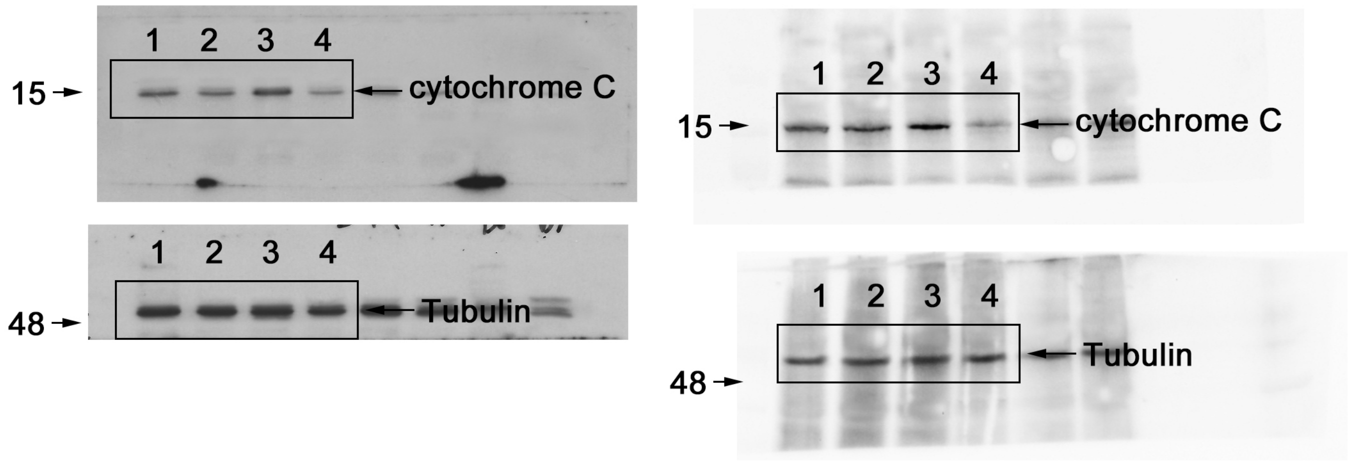

For right panel

#2 **#3**

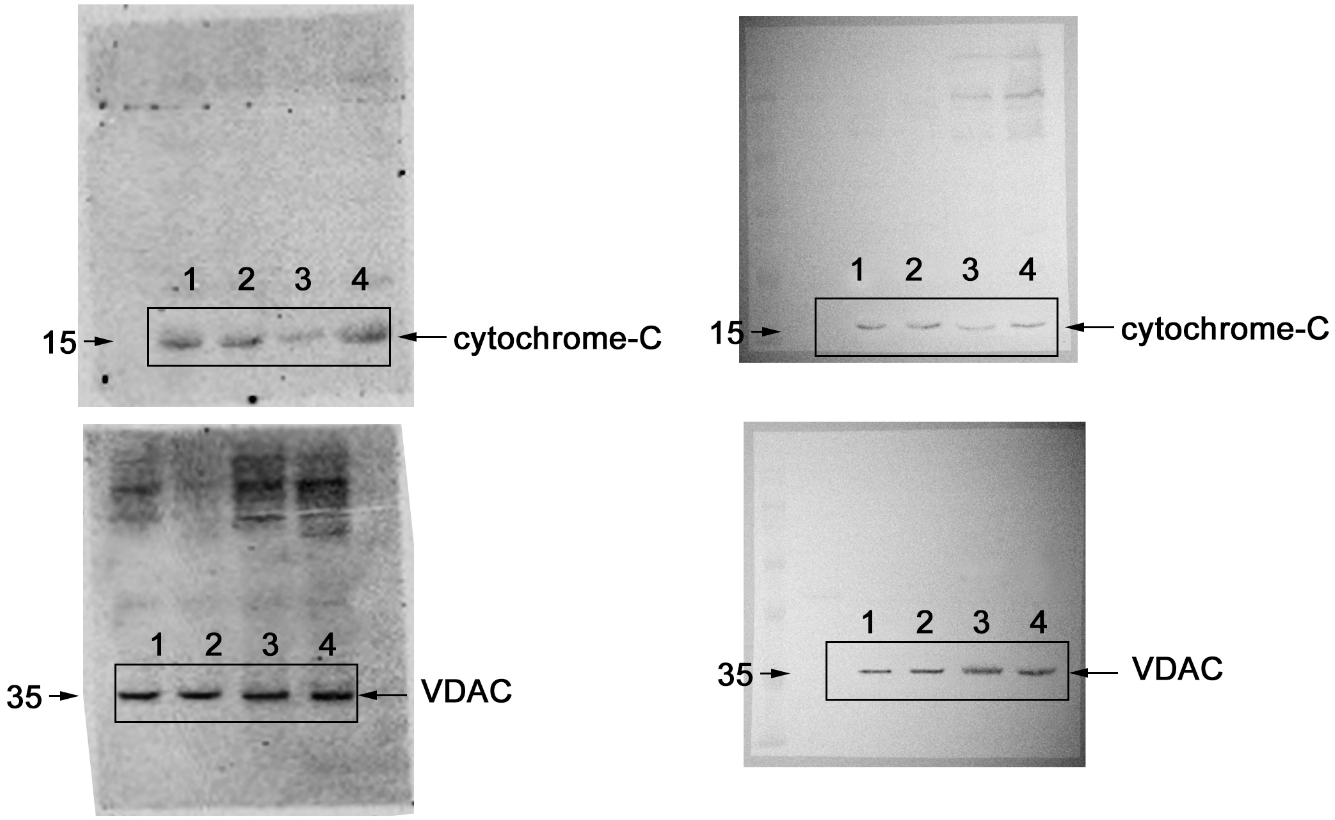

Supplement: Supplementary file 1 — Supplementary Information. [file 41598_2020_80520_MOESM1_ESM.pdf]
